# Supplementary material for: Association between stringency of lockdown measures and emergency department visits during the COVID-19 pandemic: A Dutch multicentre study
Source: PLoS One. 2024 May 21;19(5):e0303859. doi: 10.1371/journal.pone.0303859 (PMC11108187; doi:10.1371/journal.pone.0303859)
Supplement: S1 Table — H = Hospital, ICU = Intensive Care Unit, MCU = Medium Care Unit, CCU = Cardiac Care Unit, GP = general practitioner/family doctor, PCI = Percutaneous Coronary Intervention. (DOCX) [file pone.0303859.s001.docx]

**Supporting information**

**S1 Table. Hospital characteristics.**

|  | H1 | H2 | H3 | H4 | H5 |
| --- | --- | --- | --- | --- | --- |
| Type of hospital | University | Teaching hospital | Teaching hospital | University | Teaching hospital |
| Location in the Netherlands | Middle | Middle | South | Middle | North |
| Trauma Center level | 1 | 2 | 2 | 1 | 2 |
| ICU 24/7 | Yes | No | No | Yes | No |
| MCU | Yes | No | No | Yes | No |
| CCU | No | Yes | Yes | Yes | Yes |
| In hospital GP | Yes | Yes | Yes | No | No |
| PCI Center | Yes | Yes | Yes | Yes | Yes |

H=Hospital, ICU=Intensive Care Unit, MCU=Medium Care Unit, CCU=Cardiac Care Unit, GP=general practitioner/family doctor, PCI=Percutaneous Coronary Intervention
