# Supplementary material for: Association between stringency of lockdown measures and emergency department visits during the COVID-19 pandemic: A Dutch multicentre study
Source: PLoS One. 2024 May 21;19(5):e0303859. doi: 10.1371/journal.pone.0303859 (PMC11108187; doi:10.1371/journal.pone.0303859)
Supplement: Attachment — Submitted filename: Response_to_the_reviewers_25032024.docx [file pone.0303859.s002.docx]

**Response to the reviewers**

**Editors' comments**

-Please carefully address all the issues highlighted by the referees. In particular, I believe that the public health/disaster medicine side of discussion has much room to be improved.

Answer: We thank the editor for this comment. We agree on this point and added text on this matter to the Discussion section. (lines 305-310)

-*Journal Requirements:*

When submitting your revision, we need you to address these additional requirements.

1. Please ensure that your manuscript meets PLOS ONE's style requirements, including those for file naming.

Answer: The manuscript and figures/tables were adjusted according to PLOS ONE’s requirements as stated in the templates.

2. We note that you have indicated that there are restrictions to data sharing for this study. For studies involving human research participant data or other sensitive data, we encourage authors to share de-identified or anonymized data. However, when data cannot be publicly shared for ethical reasons, we allow authors to make their data sets available upon request.

Before we proceed with your manuscript, please address the following prompts:

a) If there are ethical or legal restrictions on sharing a de-identified data set, please explain them in detail (e.g., data contain potentially identifying or sensitive patient information, data are owned by a third-party organization, etc.) and who has imposed them (e.g., a Research Ethics Committee or Institutional Review Board, etc.). Please also provide contact information for a data access committee, ethics committee, or other institutional body to which data requests may be sent.

Answer: There are restrictions to data sharing for this study; see our separate statement on data sharing for this study.

3. Please include a separate caption for each figure in your manuscript.

Answer: a separate caption was made for each figure in the text.

4. Please include your tables as part of your main manuscript and remove the individual files. Please note that supplementary tables (should remain/ be uploaded) as separate ""supporting information"" files

Answer: tables were included in the main text. Supplementary was changed into supporting.

5. Please include captions for your Supporting Information files at the end of your manuscript, and update any in-text citations to match accordingly. Please see our Supporting Information guidelines for more information.

Answer: Caption for supporting information was added at the end of the main file. And the in-text citation was updated according the templates.

**Reviewers' comments**

**Reviewer #1:**

This is a retrospective, multicenter study in 5 Dutch hospitals on the association between ED utilization and the stringency of lockdown measures. Although numerous publications have emerged on ED utilization patterns during the pandemic, this manuscript is interesting because it assesses the association with the OSI index. However, there are some minor concerns and the English language could be improved throughout the manuscript.

GENERAL
The English language could be improved throughout the manuscript, particularly with regards to grammar and style. Some sentences are a little bit odd.

Answer: the authors thank the reviewer for his/her suggestions to improve the manuscript. Regarding the English language, the authors believe that grammar and style is adequate.

The suggestions below were changed in the text. If the reviewer thinks the grammar and style is insufficient, could the reviewer please specify which sentences are odd and need improvement?

ABSTRACT
- I would refrain from u- sing "usual" healthcare; consider "regular" instead

Answer: this was changed in the text accordingly (line 24)

- You state that little is known about the influence of lockdown on ED visits. This is not true and has been extensively studied. You are right about that little is known about the association of ED utilization and OSI.

Answer: we thank the reviewer for this comment. Changes were made in the abstract to make this point clear (line 27-28)

- The number of ED visits "have": should be "has"

Answer: this change was made in the abstract.

- I think there is a better wording than "tool" for OSI; maybe indicator is better (also in main article discussion and conclusion)

Answer: ‘tool’ was changed into ‘indicator’ in the main text (line 44, 338, 346)

INTRODUCTION
- COVID-19 is the disease that is caused by SARS-CoV-2, so this is not exactly the same.

Answer: Covid19 was changed into SARS-CoV-2 in line 52

- Preventative measures do not only represent lockdown measures (such as social distancing, increased hygiene measures, etc), so this part should be rephrased. It would also be good to underline that there is a great variety in lockdown; in the netherlands there never was a lockdown as stringent as in China. So consider to explain a bit more what the (first) lockdown looked like.
- Line 81-82: consider to use the word reference years instead of the two prior years.

Answer: We thank the reviewer for this important comment. We changed and rephrased the introduction to improve on this point. See lines 53-66

METHODS
no specific comments

RESULTS
no specific comments

DISCUSSION
- Good discussion of the present literature
- Line 224: I doubt if vaccination development played a role at that time, because they were not available yet.

Answer: thanks to the reviewer for this point. Although vaccinations were not available yet, there could be a psychological effect of knowing the these vaccins were developed and on the market soon. The authors suggest to leave this part unchanged.

- Line 246: consider using ED mortality instead of deceases

Answer: this was changed accordingly (line 283)

**Reviewer #2:**

This study depicts the ED fluxes of patients during the first part of the COVID-19 pandemics, discussing some useful insights for future public health measures.
I think the paper is balanced in its sections; some minor suggestions here:

- comparison 2018+2019 vs 2020 as primary aim but not present in the abstract; maybe just worth mentioning it.

Answer: we thank to reviewer for this comment. This was added in the abstract (line 33 34)

- maybe internal medicine visits preserved an increased trend because only complex cases (usually managed by IM, especially if elderly and if geriatrics not present) bypassed the patients' fear to access EDs.

Answer: We thank the reviewer for this interesting point. Although this could be an explanation, we don’t have data or references to support this suggestion. Also, especially in the academic hospital, all specialties have their high complex cases, and this argument would also apply to other specialties. Also, our data actually show a decrease in elderly patients.

Although we think this is an interesting point, we suggest to leave to conclusions about this matter unchanged in the text.

- relationships between higher ORI and negative consequences can be speculated; e.g. higher ORI could mean higher mortality or excess mortality; similar to the choice of shifting from conventional to contingency to crisis level of healthcare [ DOI: 10.1097/DMP.0b013e31819f1ae2 ; 10.1056/CAT.20.0384 ] so a message for stakeholders: you can choose to apply stricter measures, but expect consequences on population health.

Answer: Good point. We made changes in the discussion section and added these important references. (line 303-310)

Thank you for letting me review this paper.
